# Human‐Impacted Natural Ecosystems Drive Climate Warming

**DOI:** 10.1111/gcb.70449

**Published:** 2025-08-21

**Authors:** Ülo Mander, Jaan Pärn, Mikk Espenberg, Josep Peñuelas

**Affiliations:** ^1^ Department of Geography Institute of Ecology and Earth Sciences, University of Tartu Tartu Estonia; ^2^ Global Ecology Unit CREAF‐CSIC‐UAB Barcelona Catalonia Spain

**Keywords:** anthro‐natural ecosystems, carbon budget, climate change, droughts, peatlands, soil moisture

## Abstract

Current greenhouse gas budgets do not account for most indirect anthropogenic impacts. In this perspective, we call for attention to greenhouse gas fluxes from human‐impacted natural ecosystems and their mitigation measures. The article highlights the increasing greenhouse gas (GHG) emissions from natural ecosystems, including CO_2_, CH_4_, and N_2_O. These emissions are becoming significant drivers of global warming, surpassing those from fossil fuel combustion. We introduce the concept of “anthro‐natural emissions” on the example of peatlands, referring to emissions from natural ecosystems indirectly impacted by human activities. The concept helps bridge the gap between natural and anthropogenic impacts, providing a more comprehensive understanding of GHG emissions. Anthro‐natural emissions are expected to rise as climate warming progresses, contributing to the overall GHG balance. Peatlands, which store approximately 30% of the world's soil carbon, are under increasing pressure from climate warming and human activities. The article emphasizes the importance of addressing both natural and human‐impacted ecosystems to mitigate climate change effectively. Increasingly frequent droughts are identified as a major threat to global terrestrial ecosystems, particularly wetlands. The drying of wetlands challenges their capacity to act as carbon sinks and alters their roles in climate regulation. The insights provided are essential for developing effective adaptation strategies relying on soil carbon sequestration as a long‐term solution against climate warming. According to our study, the proportion of natural, anthro‐natural, and directly disturbed peatlands is approximately 40–20–40, and the ratio is increasing towards anthro‐natural peatlands. We highlight a change of paradigm for assessing the importance of different GHG sources. Further, it highlights the need for conservation and restoration of peatlands and renaturalization of forest ecosystems.

## Graphical abstract

Current greenhouse gas budgets do not account for most indirect anthropogenic impacts. In this perspective, we call for attention on anthro‐natural greenhouse gas (GHG) fluxes and their mitigation measures. We introduce the concept of anthro‐natural emissions (AnE) on the example of peatlands, referring to emissions from natural ecosystems indirectly impacted by human activities. The concept helps bridge the gap between natural and anthropogenic impacts, providing a more comprehensive understanding of GHG emissions. AnE are expected to rise as climate warming progresses, contributing to the overall GHG balance. We highlight a change of paradigm for assessing the importance of different GHG sources.

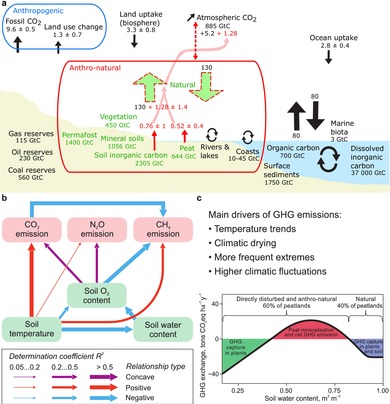

## 1 Introduction: Greenhouse Gas Emissions From Land Ecosystems on the Rise

**FIGURE 1:**
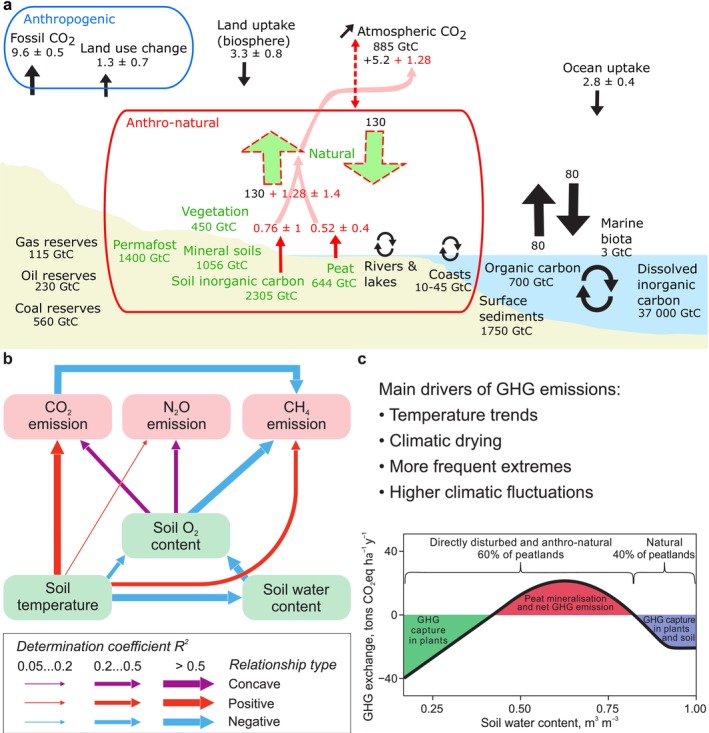
Anthropogenic impacts on global GHG cycles of terrestrial ecosystems. (a) Global carbon cycle in 2014–2023 (GtCO_2_ years^−1^). Reconceptualised from Friedlingstein et al. (2025). Additional carbon fluxes from anthro‐natural ecosystems marked by red color are based on peat losses (Leifeld and Menichetti 2017) and soil inorganic carbon losses (Huang et al. 2024) and potential fluxes from land vegetation (Erb et al. 2018). Uncertain carbon fluxes mainly from anthro‐natural ecosystems are indicated by red dotted lines. (b) Conceptual network of causal relationships between key environmental factors and emissions of three main greenhouse gases in global wetlands. *R* ^2^ values from Leifeld and Menichetti (2017), Pärn et al. (2018, 2025), Quan et al. (2019). (c) Theoretical GWP‐based CO_2_eq GHG budget of open peatlands along the soil water content gradient. In natural peatlands CH_4_ emission is high but does not exceed net CO_2_ removal. In dry section, peat mineralization is high and most probably no peat will remain. Adapted from Pärn et al. (2025).

Changes in land use, material cycling, and the energy balance of natural ecosystems due to climate warming have themselves become driving forces behind the continued rise in global temperatures. This is increasing the persistent rise of carbon dioxide (CO_2_) concentrations in the Earth's atmosphere, despite regulations on fossil fuel combustion. Gross CO_2_ emissions from terrestrial ecosystems are higher than those from fossil fuel burning (IPCC 2021; Figure 1A). Furthermore, in recent years, natural ecosystems are increasingly emitting not only CO_2_ but also other potent GHGs—methane (CH_4_) and nitrous oxide (N_2_O)—which are respectively 28 and 265 times more effective at generating the greenhouse effect than CO_2_ (IPCC 2021). Thus, a bias in the greenhouse gas (GHG) balance of natural terrestrial ecosystems toward emissions can significantly impact the overall budget, even more than restrictions on fossil fuel use (Friedlingstein et al. 2025). Therefore, while limiting fossil fuel use of course remains crucial for preventing greenhouse gas emissions, addressing both natural and human‐impacted ecosystems is also needed.

The proportion of emissions from natural ecosystems, indirectly influenced by human activity—termed “anthro‐natural emissions”—is expected to rise as climate warming progresses (Figure 1a). For instance, GHG emissions from peatlands, containing ca 30% of soil carbon (Leifeld and Menichetti 2017), are facing increasing anthro‐natural pressures (e.g., human‐induced climate warming) that will accelerate future climate warming (Figure 1b,c).

## 2 Anthro‐Natural Ecosystems for Predicting Impacts of Global Change

We define natural ecosystems as those that are hypothetically free from both direct and indirect human impacts (Erb et al. 2018). Direct impacts refer to human activities since the start of extensive agriculture and forestry. Indirect impacts on terrestrial ecosystems, including climate change‐induced positive feedbacks on carbon balance, started from the Industrial Revolution in the 1750s. We define anthro‐natural fluxes as indirectly human‐impacted fluxes, including positive feedbacks of climate change. The Industrial Revolution caused significant anthropogenic GHG emissions from fossil fuels and land use. In addition to their direct effect, positive feedbacks from the anthropogenic climate change induced a new dimension—anthro‐natural emissions. The proportion of anthro‐natural emissions is increasing and, based on wetland studies, currently reaches up to 20% of ecosystems. Anthro‐natural emissions will influence the Earth's climate over the coming centuries. For example, in a boreal forest, increased respiration rates driven by rising temperatures (due to anthropogenic GHG emissions) would be considered anthro‐natural emissions. While respiration is a natural process, any increase above baseline levels due to climate change is attributable to indirect human impacts, i.e., anthro‐natural impacts. The additional thermal energy will increase the rate of environmental change and variability, thereby accelerating global warming and GHG fluxes (Friedlingstein et al. 2025). Current GHG budgets are calculated as 10‐year averages on an annual timescale (Figure 1a), which is much shorter than the timescales required for many underlying phenomena, such as peat formation or loss. Carbon budgets for mitigation measures should reflect natural timescales, focusing on carbon sequestration in soils rather than on annual CO_2_ exchange rates between the atmosphere and ecosystems.

Climate warming will exacerbate the acidification of soils, adding an annual CO_2_ emission of 0.76 ± 1 GtC to the currently reported emissions from soil inorganic carbon (under the SSP5‐8.5‐Low‐Ambition‐N policy scenario; IPCC 2021; Figure 1a). There are major gaps in the assessment of carbon fluxes and potential stocks under different restoration scenarios. For instance, potential land vegetation—natural forest, shrubland, and grassland—would store around 916 Gt versus the current 450 Gt (Erb et al. 2018) which is likely a major reason for uncertainty in the terrestrial carbon budget (Figure 1a). Beyond the direct effects of tree cutting, the choice of tree species (e.g., conifer vs. deciduous) influences a forest's surface albedo, with impacts on radiation, moisture, and carbon balances (Naudts et al. 2016). Climatic drying across forest and shrubland ecosystems is a global trend that will contribute to increased tree mortality, forest and peatland fires, and oxygen influx into wetland soils, resulting in further GHG emissions (Pärn et al. 2025). Oxygen‐induced reduction of CH_4_ emissions from wetlands may be offset by CO_2_ and N_2_O emissions from drying. Additionally, new land areas are being released from permafrost, giving rise to little‐known ecosystems. To fill these knowledge gaps with more accurate models, more measurements are needed on GHG fluxes in natural ecosystems. We call for future studies that define natural thresholds of ecosystem GHG balances and distinguish positive feedbacks of climate change. The main focus should be on studies of long‐term carbon stock dynamics in ecosystems. Our wetlands‐based approach, presented in this paper, can be considered a first attempt toward such future studies (Figure 1c).

Therefore, potential mitigation strategies should include the conservation and restoration of peatlands, and renaturalization of current and former forest, shrubland, and grassland ecosystems (Erb et al. 2018). These efforts must be based on climate‐aware mixtures of tree species (Xu et al. 2024). The assessment of carbon fluxes from anthro‐natural ecosystems must be included in IPCC reports. Climate‐smart management and restoration strategies should become a natural part of all land policy decisions.

## 3 Anthro‐Natural Wetlands

We focus on peatlands and other wetland ecosystems in this opinion piece because they are among the most carbon‐dense ecosystems globally, making their response to anthro‐natural emissions particularly critical for the climate system. Furthermore, wetlands have been the subject of intensive research, providing a strong empirical basis for illustrating the concept of anthro‐natural emissions. While the concept applies to other ecosystems, we believe focusing on wetlands provides a clear and well‐supported example.

As a paradigmatic example of anthro‐natural ecosystems, wetlands, especially peatlands, are among the most threatened ones. Wetlands are important stores of soil organic carbon, but their existence is largely dependent on their low soil oxygen content (Figure 1b,c). This critical condition is severely threatened by drainage, climate change (increasing droughts), fires, and groundwater extraction. It is estimated that up to 13% of the Earth's peatlands have been lost in the last century. Unaccounted disturbances may increase the proportion of disturbed peatlands to nearly half (Mander et al. 2025).

Recent global studies of aerobic and anaerobic microbial processes involved in the nitrogen cycle reveal that nearly all studied marshes, including natural ones, have experienced drying for varying durations. This is corroborated by the presence of aerobic microbes in natural wetland soils (Bahram et al. 2022). The drying of most natural marshes and swamps is likely climate‐induced, driven by more frequent and severe droughts. Anthro‐natural impacts have decreased the importance of CH_4_ emissions from wetlands and the increasing amount of CO_2_ emissions (Mander et al. 2025). Peatlands store approximately 644 GtC, which, under anthro‐natural changes, may release 0.46 ± 0.4 GtC annually (Erb et al. 2018; Figure 1a). Carbon and nitrogen stocks in other wetlands are at similarly high risk (Figure 1b,c). Additionally, previously frozen permafrost areas are melting, leading to the formation of new wetlands in the coming decades (Turetsky et al. 2020). The oxygen levels influencing N_2_O formation in these future wetlands are virtually unknown, while N_2_O emissions from drying may exceed any reductions in CH_4_ emissions. Figure 1b,c, provide a framework to predict the greenhouse gas emissions from future wetlands. We highlight the combined impact of soil water and soil oxygen content on the GHGs, raising the need to focus on drought and flooding events and hotspots in model predictions. We acknowledge that wetlands are natural sources of CH_4_. However, our focus is on how climate change is altering the GHG balance in wetlands, specifically the increase in CO_2_ emissions and potentially a decrease in the relative importance of CH_4_ emissions, leading to a net positive feedback on climate warming. Figure 1c shows the theoretical relationship between the soil water content gradient and the global warming potential‐based CO_2_eq GHG in wetlands, exemplified by open peatlands. The proportion of natural, anthro‐natural, and directly disturbed peatlands is approximately 40–20–40 (Pärn et al. 2025) but the ratio is increasing towards anthro‐natural peatlands (Figure 1c). A future research and development challenge is to find quantifiable metrics to distinguish the natural and anthro‐natural ecosystems. We exemplify the challenge with the case of peatlands, which are mainly controlled by the water regime (Figure 1c).

Rewetting is a crucial step for the conservation and sequestration of carbon in previously drained peatlands, potentially transforming them into natural mires or swamp forests within decades (Mander et al. 2025). Therefore, the GWP of greenhouse gases should be considered to avoid decisions based solely on short‐term benefits, which may overlook their long‐term positive cooling effects on the climate. Rewetting abandoned peatlands for management or conservation purposes is the most viable approach for long‐term carbon sequestration in the peat. In contrast, afforestation efforts combined with ongoing peatland drainage may offer short‐term economic benefits but will ultimately lead to carbon losses in the long term (Mander et al. 2024). In the future, the inclusion of innovative methods such as integrated metagenomic, metatranscriptomic, high‐resolution mass spectrometric, and isotopic techniques should be harnessed for a better understanding of anthro‐natural GHG emissions.

## 4 Conclusions

In summary, we introduce the concept of “anthro‐natural” for ecosystems, with a focus on wetlands. These ecosystems, although deemed natural, are indirectly influenced by human activities and global warming. We have positioned these ecosystems as primary drivers of ongoing climate change, strongly warranting the inclusion of their CO_2_ emissions in global carbon budgets. Our findings underscore a feedback loop where global warming intensifies itself, as warming conditions further exacerbate greenhouse gas emissions from the affected anthro‐natural ecosystems. Finally, we have identified increasingly frequent droughts as a major threat to global terrestrial ecosystems, particularly focusing on wetlands as a paradigmatic example. These droughts challenge the capacity of wetlands to act as carbon sinks and alter their roles in climate regulation. These insights emphasize the urgent need for effective adaptation strategies that address impacts and safeguard the critical role of anthro‐natural ecosystems in the mitigation of climate change.

Quantifying the anthro‐natural emissions requires establishing a baseline for GHG fluxes and carbon stocks in the absence of climate change. This can be achieved through a combination of:

- *Historical data analysis*: Examining long‐term trends in GHG fluxes and carbon stocks to identify deviations from natural variability.
- *Process‐based modeling*: Using ecosystem models to simulate GHG fluxes under different climate scenarios, including a hypothetical scenario without anthropogenic climate change.
- *Attribution studies*: Employing statistical methods to attribute observed changes in GHG fluxes to specific drivers, including climate change and impacts of indirect human activities, thus distinguishing anthro‐natural fluxes.

## Author Contributions

**Ülo Mander:** conceptualization, funding acquisition, writing – original draft, writing – review and editing. **Jaan Pärn:** conceptualization, visualization, writing – review and editing. **Mikk Espenberg:** conceptualization, visualization, writing – review and editing. **Josep Peñuelas:** validation, writing – review and editing.

## Conflicts of Interest

The authors declare no conflicts of interest.

## Data Availability

The authors have nothing to report.
